# Editorial: Novel Targets and Biomarkers in Solid Tumors

**DOI:** 10.3389/fphar.2019.00828

**Published:** 2019-07-26

**Authors:** Zhi Shi, Hui-Qin Guo, Pascale A. Cohen, Dong-Hua Yang

**Affiliations:** ^1^Department of Cell Biology & Institute of Biomedicine, National Engineering Research Center of Genetic Medicine, Guangdong Provincial Key Laboratory of Bioengineering Medicine, College of Life Science and Technology, Jinan University, Guangzhou, China; ^2^Department of Thoracic Surgery, Beijing Sijitan Hospital, Capital Medical University, Beijing, China; ^3^Univ Lyon, Université Claude Bernard Lyon 1, INSERM U1052, CNRS 5286, Centre de Recherche en Cancérologie de Lyon, Lyon, France; ^4^Department of Pharmaceutical Sciences, College of Pharmacy and Health Sciences, St. John’s University, Queens, NY, United States

**Keywords:** solid tumor, target, biomarker, drug development, therapeutics

This research topic “Novel Targets and Biomarkers in Solid Tumors” consists of 29 articles contributed by more than 245 authors in the fields of cancer pharmacology and therapeutics. The topic collects the most relevant research in fast emerging areas of clinical molecular diagnostics, drug development, and targeting diverse signaling pathways involved in tumorigenesis and development. Our aim was to generate a collaborative discussion contributing to the future direction of pharmaceutical drug development and therapeutic options.

Transcription factors, tyrosine kinase receptors, and enzymes are closely related to tumor cell growth, proliferation, apoptosis, invasion, and metastasis*.* Vendrell et al. indicated that overexpression of the ZNF217 transcription factor was predictive of clinical response to neoadjuvant endocrine therapy (ET) in postmenopausal ER-positive (ER+) breast cancer patients. Supporting Vendrell’s group observations, Cohen et al. found that the expression of ZNF217 was predictive of the Oncotype DX^®^ (ODX) Recurrence Score in ER+ breast cancers. Liu et al. provided evidence that high expression of the transcription factor SOX2 was associated with shorter overall survival and disease-free survival in patients with triple-negative breast cancer (TNBC), and inhibition of SOX2 could be a potential therapeutic strategy for TNBC. Saby et al. highlighted the relevance of low expression of DDR1, a tyrosine kinase receptor activated by collagen in the aggressiveness and the prognosis of breast carcinoma. Bilal et al. clarified that SMS1 downregulation is associated with sphingolipid metabolism reprogramming, occurs frequently in melanoma, and constitutes a poor prognosis biomarker in metastatic melanoma. Salemi et al. examined the level of MMP-9 and circulating-free DNA BRAFV600E mutations and found that they were associated with poor prognosis. Furthermore, MMP-9 might represent a promising indicator of response to BRAF inhibitors in combination with the detection of BRAFV600E mutation. Wang et al. investigated the hematological biomarkers with regard to tumor grades, IDH, age, and sex in 706 patients with gliomas. Luo et al. provided a review focusing on molecular and histological findings in hereditary diffuse gastric cancer (HDGC) syndrome and their implications for the management of CDH1 mutation carriers and the diagnosis and treatment of HDGC. Taken together, these mechanistic-based biomarkers in tumor samples might be able to predict clinical outcome, holding great promise in clinical application.

The discovery of miRNAs and LncRNAs is propelling the future advancement of biomarker development, and they also play critical roles in tumorigenesis. Lu et al. found that miR-181a is overexpressed in gastric cancer tissues and directly inhibits caprin-1 and promotes gastric cancer development. He et al. explored the miR-26-induced apoptosis and inhibited autophagy in human non-small cell lung cancer (NSCLC) cells through the TGF-β1-JNK signaling pathway, suggesting that miR-26 could be a potential novel target for the treatment of NSCLC. Wang et al. demonstrated that miR-3188 interacted with mTOR and FOXO1 to inhibit NSCLC cell proliferation through the mTOR-p-PI3K/AKT-c-JUN signaling pathway. Therefore, miR-3188 might be a potential target for the treatment of NSCLC. Dong et al. confirmed that upregulation of lncRNA NR-046683 serves as a prognostic biomarker and potential drug target for multiple myeloma. Zhu et al. elegantly summarized the characteristics of peptides/proteins that have recently been identified as putative ncRNA translation products and their outlook for small-molecule peptide drugs, drug targets, and biomarkers. Moreover, Luo et al. summarized currently utilized biomarkers in some of the commonly known endocrine tumors, as well as future research directions.

Novel targets and biomarkers are essential components in drug developments and treatments, particularly in this era of targeted therapies. Tremendous efforts are being made to interpret the mechanisms of cancer development with the aim of discovery of novel drugs. Zhao et al. confirmed that Y6, a new epigallocatechin gallate derivative synthesized by their group, inhibited the transport activity of ABCG2. Their results showed that Y6 may potentially be a novel reversal agent in ABCG2-positive drug-resistant cancers. Ji et al. showed that VS-4718, a tyrosine kinase inhibitor targeting focal adhesion kinase (FAK), interacted with the substrate-binding sites of both ABCB1 and ABCG2 through docking study, suggesting that VS-4718 may affect the activity of ABCB1 and ABCG2 competitively. Additionally, Zhang et al. illustrated the reversal effect of olmutinib on ABCG2-mediated MDR cells. Collectively, these findings provided a novel insight into multi-drug resistance in cancer treatment. Next, Wang et al. investigated the anti-tumor effect of DT-13 on human prostate cancer cells and the underlying mechanism. Lu et al. reported the anti-tumor effect and internal molecular mechanism of ORY-1001 in KDM1A-positive lung cancer cells. Vollaire et al. demonstrated the LDN-193189 compound, a potent inhibitor of the BMP type I receptor, might affect the interaction between breast cancer cells and the bone environment, favoring the emergence and development of multiple metastases *in vivo*. Yuan et al. proved that inhibition of WEE1 by MK-1775 could exert the anti-tumor effects on laryngeal squamous cell carcinoma cells *in vivo* and *in vitro.* Finally, the review by Qin et al. presented a brief introduction on the molecular mechanisms of PARP14 as a novel drug target for several cancers, and potential PARP inhibitor-associated adverse effects are discussed.

The rapid emerging high-throughput sequencing and bioinformatics analysis technologies in gene, as well as the openness of a variety of tumor-related databases, facilitate the rapid elucidation of the intrinsic molecular mechanisms of cancer, and the screening of targets for the development of clinically effective anti-tumor drugs and biomarkers offers great opportunities. Suhaimi et al. investigated the gene mutation profile in ER-positive and -negative endometrioid endometrial cancer (EEC) through next-generation sequencing (NGS) and further elucidate the role of WHSC1 mutations in this cancer. The research method of NGS could lead to a better understanding of the biological mechanisms of cancer and may ultimately result in improvement of treatment options and patient prognosis. Menyhárt et al. identified mutations associated with elevated PD-L1 expression that facilitate the development of better prognostic biomarkers for gastric cancer, and might offer insight into the underlying tumor biology. Chen et al. commendably summarized the expression and prognostic value of PD-L1, as well as the relationships between PD-L1 and immune cell infiltration in glioma. The mechanisms regulating PD-L1 expression and the oncogenic roles of endogenous PD-L1 were discussed. Zhang et al. investigated the protein expression of C-MYC, BCL-2, and BCL-6 in diffuse large B-cell lymphoma and their relationship with genetic abnormalities. Overexpression of those proteins suggested the possibility of translocation. Therefore, immunohistochemical detection of C-MYC, BCL-2, and BCL-6 was useful in diagnosis and prognosis of diffuse large B-cell lymphoma. Xiang et al. performed a cross-database study to analyze the data of ERBB2^+^ gastric cancer deposited in the cancer genome atlas (TCGA), gene expression omnibus (GEO), InBio Map™, cancer cell line encyclopedia (CCLE), and cancer therapeutics response portal (CTRP), and found that a combination of ERBB2 antagonist or RARA agonist might be effective synergistic regimens for ERBB2^+^ gastric cancer. Zhang et al. explored potential biomarkers associated with the Lauren classification of gastric cancer through screening microarray datasets with information of Lauren classification in the GEO database and comparing differentially expressing genes between intestinal-type or diffuse-type gastric cancer. Chen et al. demonstrated that DEAD-Box helicase 5 interacted with transcription factor 12 and promoted the progression of osteosarcoma by stimulating cell cycle progression.

In conclusion, the “Novel Targets and Biomarkers in Solid Tumors” research topic highlights the importance of developing novel targets and biomarkers for cancer diagnosis and therapy.

## Author Contributions

All authors listed have made a substantial, direct, and intellectual contribution to the work, and approved it for publication.

## Funding

ZS was supported by funds from the National Key Research and Development Program of China No. 2017YFA0505104 (ZS) and the National Natural Science Foundation of China No. 81772540. PC was supported by grants from by the French Ligue Contre le Cancer (committees 42 and 71) and Agence Nationale de la Recherche, France (2011 ANR-CESA-018-01).

## Conflict of Interest Statement

The authors declare that the research was conducted in the absence of any commercial or financial relationships that could be construed as a potential conflict of interest.

